# Cryptic Transcription Mediates Repression of Subtelomeric Metal Homeostasis Genes

**DOI:** 10.1371/journal.pgen.1002163

**Published:** 2011-06-30

**Authors:** Isabelle Toesca, Camille R. Nery, Cesar F. Fernandez, Shakir Sayani, Guillaume F. Chanfreau

**Affiliations:** Department of Chemistry and Biochemistry and the Molecular Biology Institute, University of California Los Angeles, Los Angeles, California, United States of America; University of California San Francisco, United States of America

## Abstract

Nonsense-mediated mRNA decay (NMD) prevents the accumulation of transcripts bearing premature termination codons. Here we show that *Saccharomyces cerevisiae* NMD mutants accumulate 5′–extended RNAs (CD-CUTs) of many subtelomeric genes. Using the subtelomeric *ZRT1* and *FIT3* genes activated in response to zinc and iron deficiency, respectively, we show that transcription of these CD-CUTs mediates repression at the *bona fide* promoters, by preventing premature binding of RNA polymerase II in conditions of metal repletion. Expression of the main *ZRT1* CD-CUT is controlled by the histone deacetylase Rpd3p, showing that histone deacetylases can regulate expression of genes through modulation of the level of CD-CUTs. Analysis of binding of the transcriptional activator Zap1p and insertion of transcriptional terminators upstream from the Zap1p binding sites show that CD-CUT transcription or accumulation also interferes with binding of the transcriptional activator Zap1p. Consistent with this model, overexpressing Zap1p or using a constitutively active version of the Aft1p transcriptional activator rescues the induction defect of *ZRT1* and *FIT3* in NMD mutants. These results show that cryptic upstream sense transcription resulting in unstable transcripts degraded by NMD controls repression of a large number of genes located in subtelomeric regions, and in particular of many metal homeostasis genes.

## Author Summary

Precise expression of genes relies on their accurate and timely transcription and on turning off these genes when production of the proteins is not required. Our study describes that in the baker's yeast, *S. cerevisiae*, repression of many genes relies on transcription of long extended RNAs upstream from where transcription normally initiates. These long extended RNAs are degraded by a machinery that recognizes that these RNAs do not encode any functional proteins. Using genes involved in controlling the uptake of the essential elements zinc and iron, we find that transcription of these long extended non-coding RNAs represses transcription by preventing the binding of the transcriptional machinery to the normal transcriptional control elements. Our findings show that repression of transcription of many genes relies on the transcription of unstable long RNAs and that this mechanism of control is particularly prevalent for genes involved in controlling the level of metals inside cells.

## Introduction

A large fraction of eukaryotic genomes is transcribed, even in the non-coding regions (reviewed in [1]). One of the major questions that arise from these observations is to understand whether the RNAs expressed from these regions serve any functional purpose or whether they correspond to genomic noise that is ultimately routed for degradation. Transcription of non-coding (nc) RNAs nearby protein-coding genes has emerged as a means of transcriptional control (reviewed in [1], [2]). In the yeast *S.cerevisiae*, the first and best-documented example is the *SRG1* ncRNA, which regulates transcription of the *SER3* gene involved in serine metabolism through transcriptional interference [3]. While the *SRG1* ncRNA is transcribed in the sense direction upstream of its target gene, most ncRNAs found to regulate *S.cerevisiae* transcription are antisense transcripts, such as the ones described for the *PHO84*, *Ty-1* and *GAL10* loci [4]–[7], which control chromatin modification marks at these genes. However, upstream sense transcription resulting in the *ICR1* ncRNA has been found to regulate the *FLO11* gene [8].

Cryptic transcripts can also be generated through the transcription of elements that control the expression of *bona fide* protein coding genes. There is ample evidence that promoter regions are associated with bidirectional transcription in *S.cerevisiae* [2], [9]. In addition, it has been shown that enhancers are transcribed by RNA polymerase II [10]. In *S.cerevisiae*, many transcripts associated with intergenic or promoter transcription are unstable under normal conditions. They are hardly detectable in wild-type strains because of their rapid degradation by nuclear RNA turnover. Indeed, many transcripts associated with cryptic transcription are detectable only when the activity of the nuclear exosome, or that of the TRAMP-complex, which stimulates exosome activity, is inhibited [2], [11], [12]. Therefore, these transcripts have been labeled “CUTs” for Cryptic Unstable Transcripts. Most of the degradative activities targeting CUTs seem to be concentrated in the nucleus. However, the cytoplasmic exonuclease Xrn1p can degrade the antisense RNAs that regulate the *Ty-1* gene [5]. In addition, several CUTs can be degraded by the cytoplasmic degradation machinery [12]–[13], showing that the degradation of RNAs arising from transcription in non-coding regions can result from both nuclear and cytoplasmic RNA degradation pathways.

Nonsense mediated decay is an RNA surveillance mechanism that recognizes transcripts containing premature translation termination codons (PTCs; [14], [15]). This degradation system is used to prevent accumulation of aberrant mRNAs that would encode potentially toxic proteins [16], [17], but is also used for gene expression control. For example, NMD degrades alternatively or inefficiently spliced mRNAs that contain PTCs [18]–[21], and transcripts containing long 3′-UTRs [22]. In *S.cerevisiae*, NMD also controls Mg^++^ uptake by degrading the transcript encoding the major Mg^++^ transporter [23]. Previous studies using microarray analysis of *S.cerevisiae* NMD mutants have shown that NMD influences directly or indirectly the expression of many genes, including some that do not exhibit PTCs [24]–[26]. The identification of RNAs that associate with the NMD factor Upf1p by RNA pull-down has proved to be an efficient way to discriminate direct and indirect NMD targets [27]. However, many NMD targets do not accumulate to high levels in the presence of a functional NMD system, which renders this approach difficult for low expression transcripts. In this study, using tiling microarrays, we show that NMD degrades 5′-extended transcripts generated by cryptic transcription upstream from *bona fide* promoters, and that upstream transcription is responsible for the repression of metals homeostasis genes in conditions of metal repletion. These results show that NMD controls the expression of a large number of genes by modulating the expression of 5′-extended RNAs that control transcription.

## Results

### Accumulation of 5′-extended unstable RNAs of subtelomeric genes in NMD mutants

**Figure 1:**
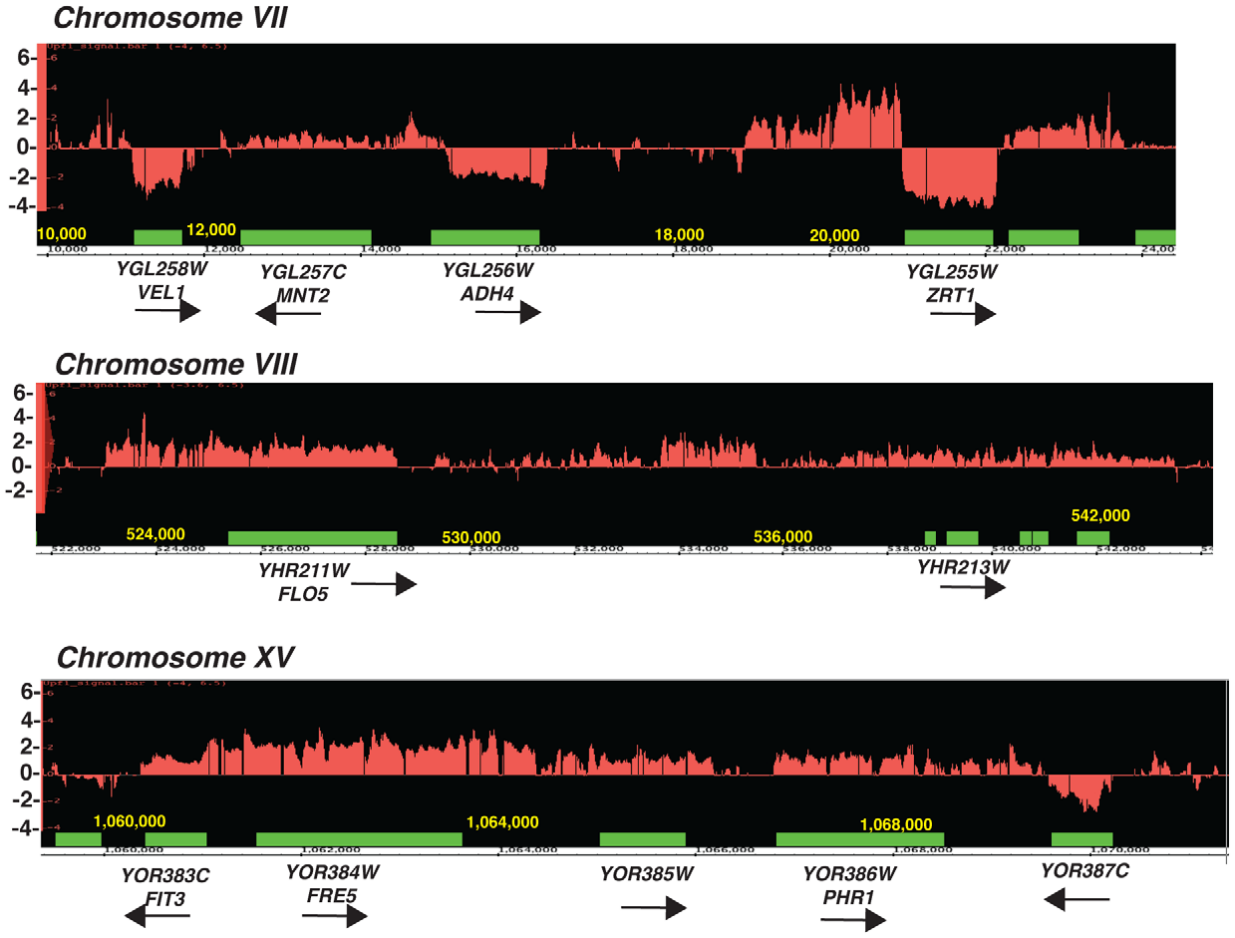
Tiling arrays profiles of the *upf1Δ* mutant relative to the wild-type strain. Green boxes represent the boundaries of open-reading frames, red the log_2_ ratio of the RNA signals detected in the *upf1* ***Δ*** mutant relative to wild-type. Arrows indicate the direction of transcription of the ORFs. Shown are three segments of three chromosomes.

**Figure 2:**
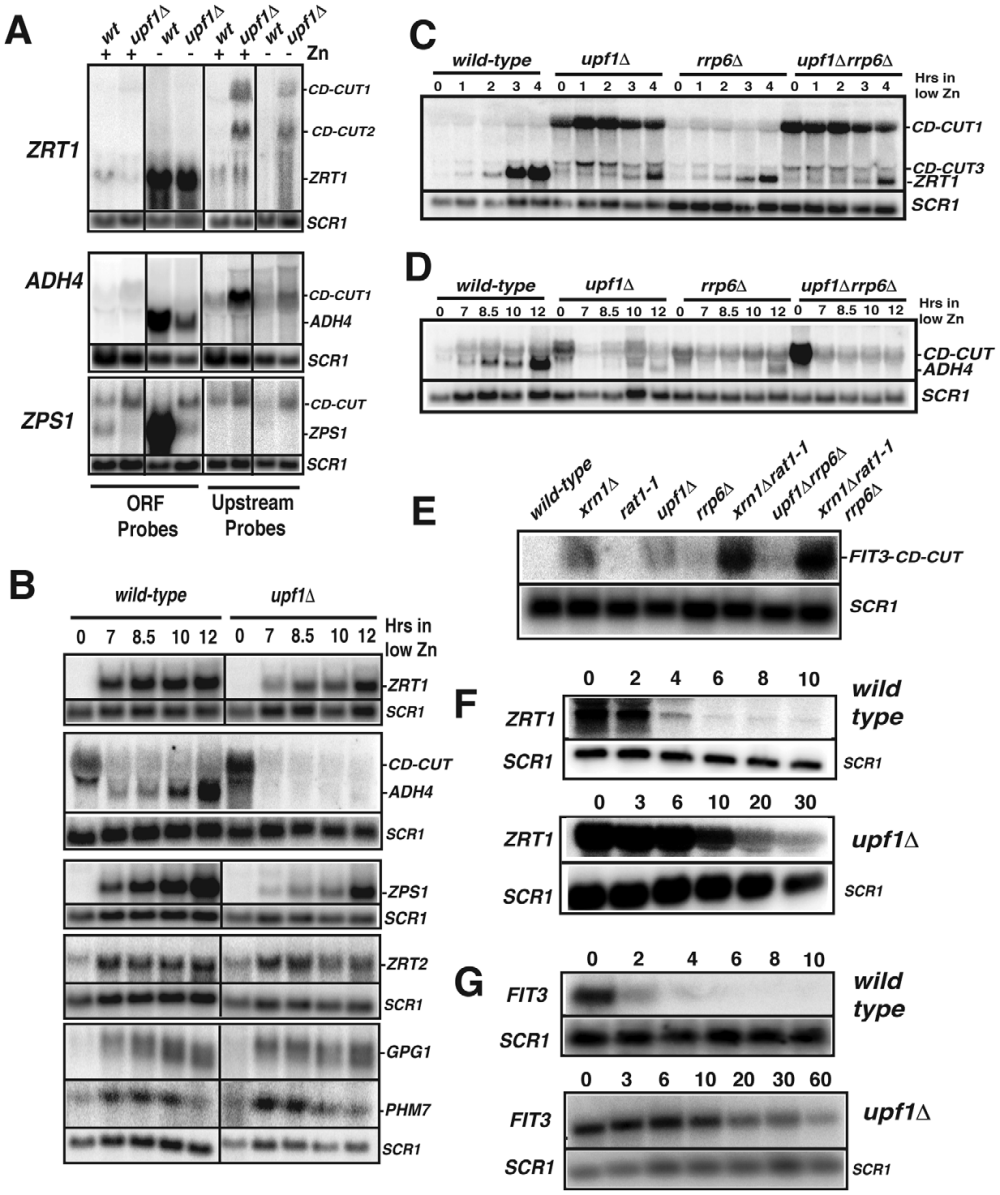
Characterization of CD-CUT of zinc and iron responsive genes. A. Northern blots of indicated genes in wild-type and *upf1Δ* strains grown in the presence (+) or absence (−) of zinc. Extended species are marked as CD-CUT1 and 2. *SCR1* was used as a loading control. B. Kinetics of induction of zinc regulon genes in wild-type and *upf1Δ* strains after a shift to a medium lacking zinc. C–E. Analysis of *ZRT1*, *ADH4* and *FIT3* CD-CUT and mRNA expression in ribonuclease mutants. C. Northern analysis of *ZRT1* induction in wild-type, *upf1Δ*, *rrp6Δ* and *upf1Δrrp6Δ* prior to and after a shift into low zinc medium. D, as in A for *ADH4*. E, northern analysis of the *FIT3* CD-CUT in iron-replete conditions in the indicated strains. F–G. Analysis of *ZRT1* (F) and *FIT3* (G) CD-CUT turnover in wild-type and *upf1Δ* strains. The GAL promoter was inserted upstream from the major site of transcription initiation of the *ZRT1* or *FIT3* CD-CUTs. After growth in galactose containing medium, cells were switched to glucose-containing medium and cell aliquots were harvested at the indicated times after the switch. CD-CUT levels were analyzed by northern blots using upstream riboprobes.

We previously used tiling microarrays to show that NMD controls the degradation of inefficiently spliced *S.cerevisiae* pre-mRNAs [21]. Further analysis of these microarrays in intergenic areas revealed that many subtelomeric regions accumulated RNA signal outside and upstream from the open-reading frames (ORF) in the *upf1Δ* and *xrn1Δ* mutants. Examples shown in Figure 1 include the subtelomeric *ZRT1* gene encoding the primary zinc transporter [28], the *FIT3* gene involved in siderophore-iron transport facilitation [29], and the *FLO5* gene encoding a cell wall protein (Figure 1). Many genes located in subtelomeric regions are involved in the response to adverse growth conditions [30]. In the case of the *FIT3* and *FLO5* genes, the array profiles suggested that these species originate 5′-to the normal transcriptional start site and extend in the open-reading frame (which was confirmed by northern analysis, see below). In the case of *ADH4* and *ZRT1*, increase of signal in the upstream regions correlated with a decrease of signal in the downstream regions (Figure 1). Northern blot analysis using antisense riboprobes confirmed that these species correspond to 5′-extended transcripts originating upstream from the *bona fide* promoters and partially or completely overlapping the open-reading frame (Figure 2 and see below). Based on the array profiles, some of these species were very long, containing 5′-extensions up to several kb for *ZRT1* (Figure 1, Figure S2). In the case of *FIT3*, a previous study had mapped in detail the 5′-extended species that extends into the ORF [31]. Thus, these species are different from the short antisense CUTs transcribed divergently from the promoters [2], [9]. Accumulation of 5′-extended transcripts in NMD mutants has been reported for a few transcripts, but was interpreted as the result of transcriptional noise [27]. We hypothesized that some of these 5′-extended unstable transcripts might be used for regulatory purposes, as shown in other systems [3], [32], [33]. Because the accumulation of 5′-extended transcripts relied on the inactivation of cytoplasmic degradation pathways, we named these 5′-extended species CD-CUTs (Cytoplasmically Degraded Cryptic Unstable Transcripts).

### NMD mutants show a delay in the induction of subtelomeric metal homeostasis genes

In the case of subtelomeric genes involved in zinc metabolism (*ZRT1*, *ADH4*, *VEL1*, *YOR387C*, Figure 1; ZPS1, Figure S1), the accumulation of CD-CUTs was correlated with a decreased signal in the ORF regions, suggesting that they might play a negative role in the expression of the *bona fide* mRNAs. Interestingly, zinc regulon genes not located in subtelomeric areas, such as the one encoding the low affinity zinc transporter *ZRT2* [34] did not exhibit CD-CUTs (data not shown). We found that the growth conditions used for the microarrays (synthetic complete minimal medium) corresponded to mild zinc deficiency conditions, in which some of the zinc regulon genes were partially induced (data not shown). Therefore the decrease of signal observed in the ORF regions for *ZRT1* and *ADH4* (Figure 1) might be due to a defect in the partial induction of these genes in minimal medium. We further analyzed the expression of zinc responsive mRNAs and their corresponding CD-CUTs in wild-type and *upf1Δ* strains grown in the presence or absence of zinc, using northern blots and antisense riboprobes covering the open-reading frames (ORFs) or the upstream (UP) regions (Figure 2A). This analysis confirmed the accumulation of CD-CUTs of *ZRT1*, *ADH4* and *ZPS1* in the *upf1Δ* mutant strain. For *ZPS1*, CD-CUTs were readily detectable in the wild-type as well as in the *upf1Δ* strain, explaining why only a modest increase in upstream signal was observed for this gene on the arrays, which compare the transcripts levels of the *upf1Δ* mutant to the wild-type (Figure S1). Some CD-CUTs were detected only with the upstream riboprobes, demonstrating that they are independent from the ORFs (Figure 2A). However, most CD-CUTs were also detected using the ORF riboprobes (Figure 2A and see below), indicating that they extend through the ORFs. A detailed characterization of the *ZRT1* and *ADH4* CD-CUTs using various upstream probes and ORF probes is shown in Figure S2 and described below. ORF riboprobes detected the induction of the normal *ZPS1*, *ADH4*, and *ZRT1* mRNAs in wild-type cells grown in a medium lacking zinc (Figure 2A). However, these mRNAs were less abundant in the *upf1Δ* mutant (Figure 2A). This observation suggested that the accumulation of CD-CUTs due to NMD inactivation was deleterious to the expression of the *bona fide* mRNAs. We further characterized the expression of these genes in the *upf1Δ* mutant by monitoring their kinetics of induction (Figure 2B). This experiment showed that the *upf1Δ* mutant exhibited a delay in the induction of the subtelomeric *ZPS1*, *ZRT1*, *ADH4* (Figure 2B) and *VEL1*/*YOR387C* genes (Figure S3A). The induction defect was also observed in the *upf2Δ* and *upf3Δ* strains (Figure S3A), indicating that it is a general feature of NMD mutants. In contrast, the induction of other zinc regulon genes such as *ZRT2*, *GPG1* (*YGL121C*) and *PHM7* (*YOL084W*), which are not localized in subtelomeric regions and do not exhibit CD-CUTs (data not shown) was unaffected by Upf1p absence (Figure 2B). A previous study found that NMD can influence gene expression by controlling the level of transcriptional activators [35]. We found that the mRNA levels of the Zap1p activator (which activates zinc regulon genes) were similar in wild-type and *upf1Δ* mutants (Figure S3B), suggesting that the delay of induction in the absence of Upf1p was not due to reduced Zap1p levels. Overall these results show that the defective induction of subtelomeric zinc-responsive genes in the *upf1Δ* mutant is not due to a global deficiency in zinc sensing in this mutant, but is specific to genes that exhibit CD-CUTs. Defective induction was also observed for the *FIT3* iron depletion-responsive gene in a strain lacking Xrn1p or Upf1p activity (Figure S4). Overall these observations suggest that the presence or transcription of CD-CUTs represses the expression of many subtelomeric genes, some of which are involved in zinc or iron homeostasis.

### Long 5′-extended transcripts of subtelomeric genes are primarily targeted by NMD, while short 5′-extended transcripts are degraded by NMD and nuclear RNA degradation pathways

To further investigate the mechanisms of turnover of CD-CUTs by the different RNA degradation machineries, we analyzed the accumulation of CD-CUTs of *ZRT1* and *ADH4* in strains lacking Upf1p, the nuclear exosome component Rrp6p, or both. These two genes were chosen because the 5′-extensions found in the CD-CUTs of *ZRT1* and *ADH4* are very different in size (extension of 2 kb for *ZRT1* vs. a few hundred nucleotides for *ADH4*). This analysis showed that the major *ZRT1* CD-CUT (CD-CUT1, Figure 2C) accumulated dramatically in the *upf1Δ* strain, and to a much lesser extent in the *rrp6Δ* mutant. Accumulation of the *ZRT1* main CD-CUT-1 was not increased in the *upf1Δrrp6Δ* double mutant compared to the *upf1Δ* single mutant (Figure 2C). These observations suggest that the degradation of the longer CD-CUT of *ZRT1* relies mostly on NMD, consistent with the long upstream region lacking any extended ORF. In contrast, the short *ADH4* CD-CUT accumulated to similar levels in the *upf1Δ* and *rrp6Δ* strains (Figure 2D). Strikingly, the accumulation of this CD-CUT was dramatically increased in the *upf1Δrrp6Δ* double mutant (Figure 2D, time zero), suggesting that this CD-CUT is degraded by the cooperative action of the nuclear exosome and of NMD. Interestingly the induction of the *bona fide ADH4* mRNA was completely defective in this double mutant strain, while the single mutants were only partially delayed. The correlation between the strong accumulation of the *ADH4* CD-CUT in the *upf1Δrrp6Δ* double mutant and the severe induction defect of the *bona fide ADH4* mRNA provides further evidence for the repression of this subtelomeric gene by its CD-CUT.

The CD-CUT of the subtelomeric iron responsive gene *FIT3* was previously shown to accumulate in the *xrn1Δrat1-1* double mutant [31], raising the question of which exonuclease was primarily responsible for its degradation. We analyzed the expression of this CD-CUT in strains lacking Xrn1p, Upf1p, Rrp6p, in the *xrn1Δrat1-1* mutant strain and in other double mutant strains. This analysis revealed that the long CD-CUT of *FIT3* accumulated to the highest level in the strain lacking Xrn1p and to a lesser extent Upf1p (Figure 2E), and its accumulation was not dramatically increased by Rrp6p inactivation, in contrast to what was found for *ADH4*. Thus, based on this steady-state analysis, the long CD-CUT of *FIT3* is also primarily targeted by cytoplasmic turnover pathways that include Xrn1p and Upf1p.

### The turnover of the *ZRT1* and *FIT3* CD-CUTs is dependent on NMD

Because CD-CUTs exhibit a lack of extended ORFs, we interpreted their accumulation in the *upf1Δ* and *xrn1Δ* mutant strains as the result of a lack of degradation by NMD. Alternatively, we could not rule out that transcription of these upstream regions might be indirectly up-regulated in these mutants. To test this hypothesis, *GFP-HIS3* cassettes [36] were inserted upstream from the normal *ZRT1* or *ADH4* promoters in wild-type and *upf1Δ* strains, such that the expression of the *GFP* mRNA was under the control of the upstream regions (Figure S5A). Northern blot analysis of *GFP* inserted upstream from *ZRT1* or *ADH4* showed that this reporter transcript was expressed at similar levels in the wild-type and *upf1Δ* strains (Figure S5B). These results suggested that the accumulation of CD-CUTs in NMD mutants is not due to an increased transcription of these upstream regions, but to the absence of ORF in the 5′- extension of the CD-CUTs.

To gain further evidence that the turnover of CD-CUTs is directly dependent on NMD, we replaced the region upstream from the site of transcription initiation of the *ZRT1* and *FIT3* CD-CUTs with a galactose inducible promoter. This allowed us to measure the rate of decay of these CD-CUTs in the presence or absence of functional NMD. The kinetics of turnover of the *ZRT1* CD-CUT (Figure 2F) or of the *FIT3* CD-CUT (Figure 2G) showed that these species are much more unstable in the presence of functional NMD (t_1/2_ = 2–3 min.) than in the absence of Upf1p (t_1/2_ = 20–30 min.). Because the turnover rate of these species is strongly decreased when NMD is inactivated, we conclude that these CD-CUTs are directly targeted by NMD for their degradation.

### The main CD-CUT of *ZRT1* is activated by the histone deacetylase Rpd3p

**Figure 3:**
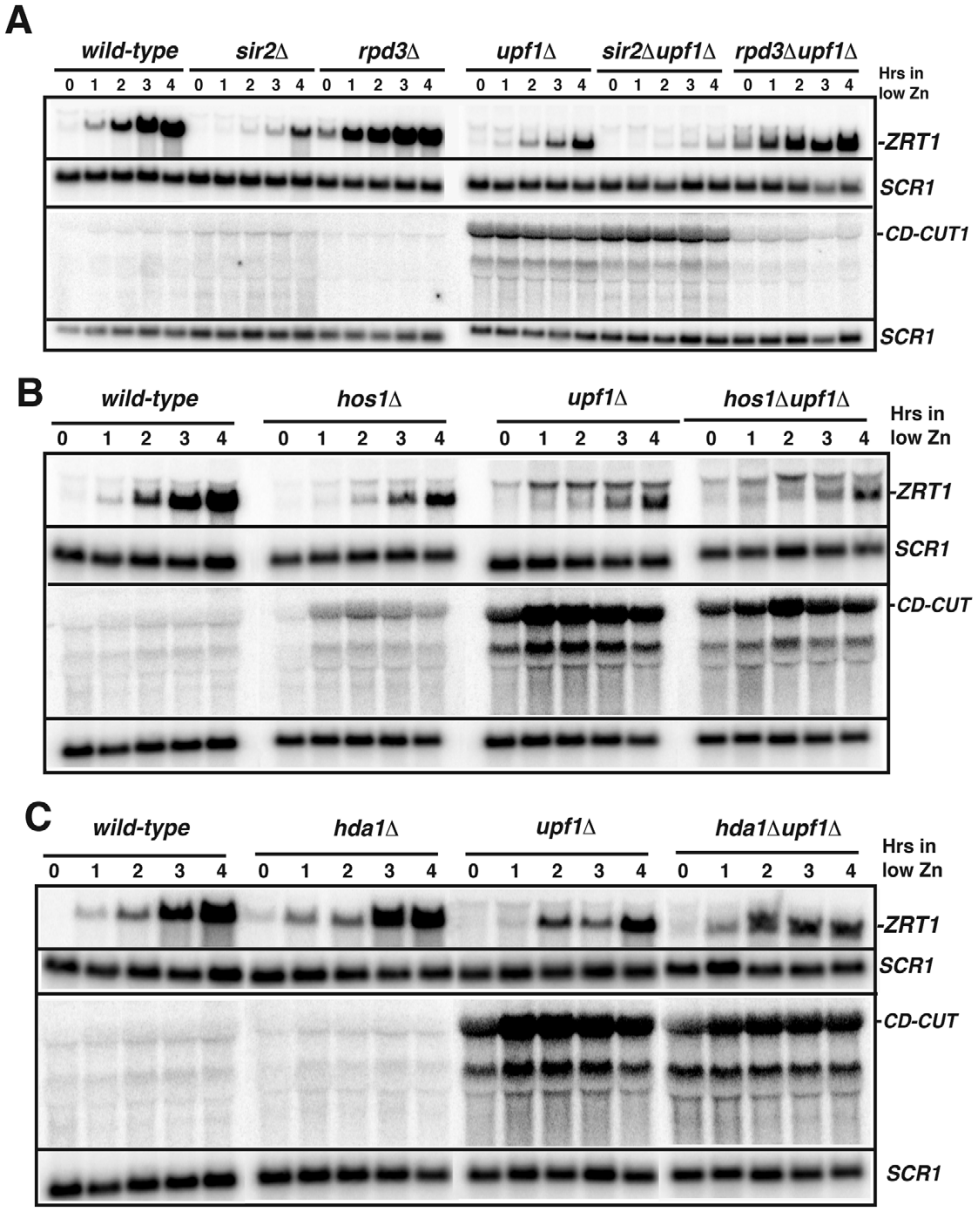
The main *ZRT1* CD-CUT is controlled by the histone deacetylase Rpd3p. A. Northern blot analysis of *ZRT1* mRNA and CD-CUTs in wild-type, *sir2Δ*, *rpd3Δ*, *upf1Δ* and *sir2Δupf1Δ* and *rpd3Δupf1Δ* deletion strains. B. Northern blot analysis of *ZRT1* mRNA and CD-CUTs in wild-type, *hos1Δ*, *upf1Δ* and *hos1Δupf1Δ* strains. C. Northern blot analysis of *ZRT1* mRNA and CD-CUTs in wild-type, *hda1Δ*, *upf1Δ* and *hda1Δupf1Δ* strains.

**Figure 4:**
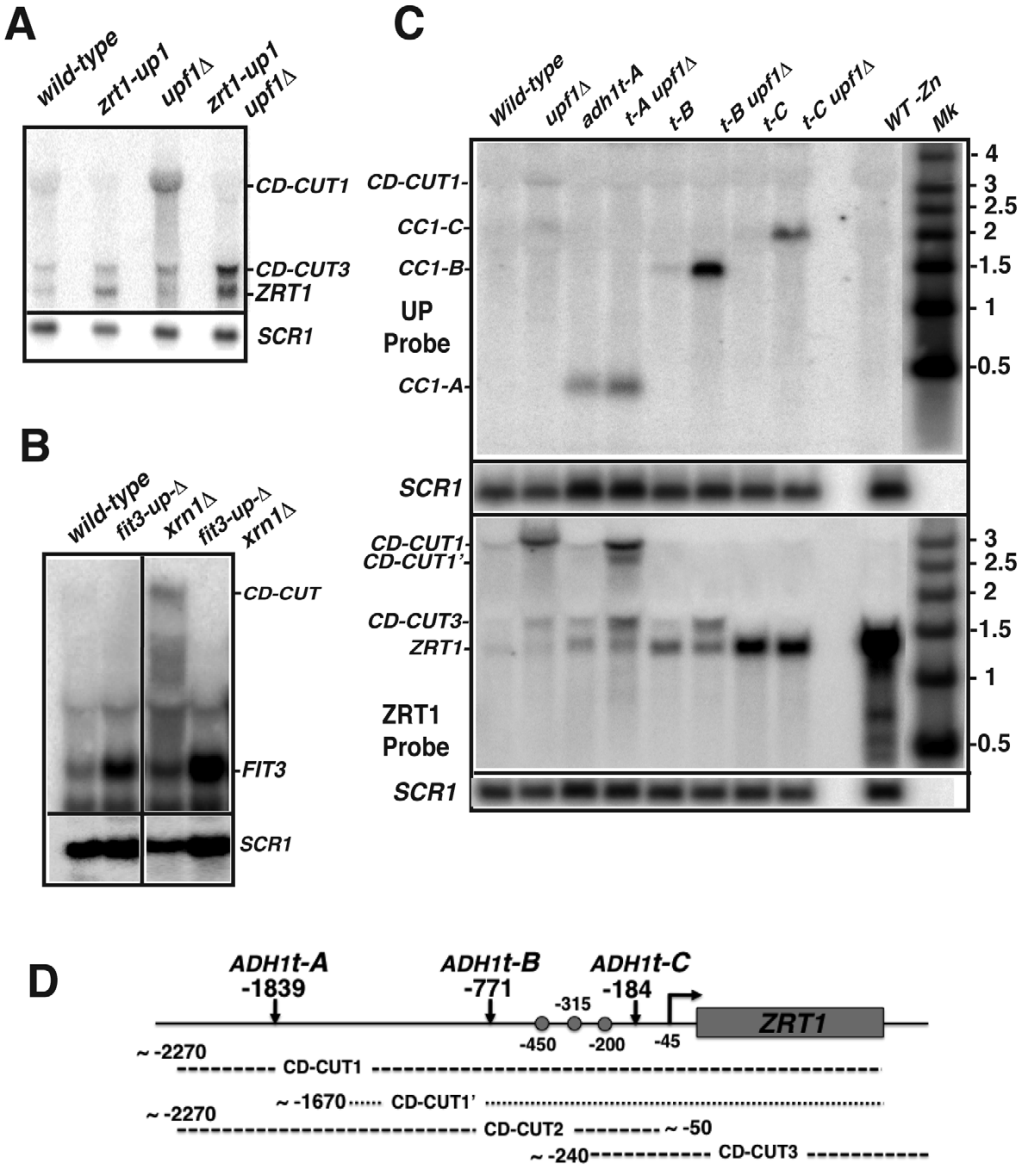
Mutations of the upstream regions and insertion of transcription terminators result in a derepression of *ZRT1* and *FIT3*. A. Effects of the insertion of a *GFP-HIS3* cassette upstream *ZRT1* (*zrt1-up1*) on *ZRT1* mRNA levels in zinc repletion conditions in wild-type and *upf1Δ* strains. *ZRT1* RNAs were assessed by northern blot using an ORF probe. B. Effects of the deletion of the region upstream *FIT3* (*fit3-upΔ*) on *FIT3* levels in iron repletion conditions in wild-type and *xrn1Δ* strains. *FIT3* RNAs were assessed by northern blot using an ORF probe. C. Effects of the insertion of *ADH1t* transcriptional terminators at various sites upstream *ZRT1* on *ZRT1* mRNA and CD-CUT levels in zinc repletion conditions in wild-type and *upf1Δ* strains. Mk is a size marker. The upper membrane was hybridized with a probe upstream from the *ZRT1* ORF, while the lower membrane was hybridized with a probe corresponding to the *ZRT1* ORF. D. *ADH1t* terminator insertion sites and schematic maps of *ZRT1* CD-CUT species. Dots represent the three major Zap1p binding sites.

Previous microarray analysis of a strain inactivated for the histone deacetylase Rpd3p showed that *ZRT1* is derepressed in the *rpd3Δ* strain and that Sir2p played a role antagonistic to Rpd3p in *ZRT1* expression [37]. To investigate whether Rpd3p or Sir2p control *ZRT1* by modulating the expression of its CD-CUT, we inactivated Upf1p in *rpd3Δ* or *sir2Δ* backgrounds and studied the induction of *ZRT1* in these strains. *ZRT1* was strongly derepressed in the *rpd3Δ* strain (Figure 3A, time zero), in agreement with previous data [37]. Strikingly, inactivation of Rpd3p in the *upf1Δ* strain completely rescued the induction defect of this NMD mutant, and resulted in a strong derepression of *ZRT1* in normal zinc conditions (Figure 3A). Inactivation of Rpd3p also resulted in the almost complete disappearance of the *ZRT1* CD-CUT observed in the *upf1Δ* strain. These results show that Rpd3p positively controls the expression of CD-CUT of *ZRT1*, and suggest that the derepression of *ZRT1* in the *rpd3Δ* mutant [37] is due to the absence of the CD-CUT. In contrast, Sir2p inactivation reduced *ZRT1* levels (Figure 3A), in agreement with the previous results [37]. Combining the *sir2Δ* deletion to the *upf1Δ* deletion exacerbated the *ZRT1* induction delay when compared to the *upf1Δ* mutant (Figure 4A), but the *sir2Δupf1Δ* mutant did not exhibit higher levels of CD-CUT than the *upf1Δ* single mutant (Figure 4A). Therefore, the negative effects of Sir2p inactivation on *ZRT1* expression are unlikely to be directly linked to its effect on the *ZRT1* CD-CUT.

To investigate the specificity of the effect observed with Rpd3p on the *ZRT1* CD-CUT levels, we performed the same genetic analysis with the Hos1p, Hda1p, Hda2p and Hda3p deacetylases. The *hos1Δ* strain showed a slight delay in the induction of *ZRT1*, correlated with an increase of CD-CUT levels, but the *hos1Δupf1Δ* double mutant showed no additive effect when combined with the *upf1Δ* deletion (Figure 3B). Neither Hda1p (Figure 3C), nor Hda2p or Hda3p (Figure S6) were found to affect *ZRT1* induction or repression. These results show that the major effect observed with Rpd3p on the *ZRT1* CD-CUT is specific to this deacetylase. We tried to corroborate these results by monitoring the presence of Rpd3p in the region 5′ to *ZRT1* but could not obtain reproducible evidence for enrichment by ChIP (data not shown). However the genetic data shown above strongly suggest that Rpd3p mediates the repression of *ZRT1* through the modulation of the transcription of the CD-CUT.

### Inactivation of the transcription of the CD-CUTs by deletion or transcriptional termination relieves *ZRT1* and *FIT3* repression and can rescue the induction defect of the *upf1Δ* mutant

If CD-CUTs are involved in the repression of the *ZRT1* gene, we predicted that the replacement of its upstream region by the *GFP*-*HIS3* coding cassette (Figure S5A) might alleviate its repression. Northern blot analysis of the strain carrying one of the insertions upstream from *ZRT1* (*zrt1-up1*; inserted 1978 to 578 nucleotides upstream from the *ZRT1* ATG; Figure S5A) showed a four-fold derepression of *ZRT1* in zinc repletion conditions, both in the wild-type and *upf1Δ* backgrounds (Figure 4A). Insertion of this cassette eliminated the detection of the main CD-CUT of *ZRT1*, with the exception of the short CD-CUT3 (Figure 4A). This insertion also partially suppressed the induction defect of the *upf1Δ* strain during zinc deficiency (Figure S5C). The kinetics of disappearance of *ZRT1* upon shifting back to zinc-containing medium was also monitored in these strains after 4 hours of induction, but we found no difference in the rate of *ZRT1* shutoff in the presence or absence of its main CD-CUT (Figure S5C). A similar derepression was observed for *FIT3* in a strain carrying a 3 Kb deletion of the region upstream of the *FIT3* gene (from −4 kb to −1 kb upstream *FIT3*; *fit3-upΔ*, Figure 4B). Analysis of the *fit3-upΔxrn1Δ* double mutant strain showed that the CD-CUTs of *FIT3* were eliminated in this double mutant, which confirmed that the derepression was due to the absence of the CD-CUT. Thus, deleting the regions encoding the CD-CUTs is sufficient to trigger derepression of the *bona fide* mRNAs, even in a wild-type context.

To provide more direct evidence that transcription of CD-CUTs is responsible for repression of the downstream promoters, we inserted the *ADH1* transcription terminator (*ADH1t*) at 3 positions upstream from *ZRT1* (*tA*: −1839, *tB*: −771 and *tC*: −184 bp; Figure 4C, 4D). If CD-CUT transcription or accumulation prevents the binding of RNA polymerase or of the transcriptional activator Zap1p, we hypothesized that terminating transcription of the CD-CUTs prior to the *ZRT1* transcriptional control elements could derepress *ZRT1* and/or rescue of the induction defect of NMD mutants. We first assessed *ZRT1* mRNA and CD-CUTs levels in these strains in normal zinc conditions (Figure 4C). A sample from a strain grown in low zinc conditions was included as a control for the *ZRT1* mRNA. Insertion of *ADH1t* at position A did not result in major *ZRT1* derepression, probably because terminating transcription at this site results in activation of an alternative CD-CUT downstream from that site (labeled CD-CUT1′. Figure 4C). However insertion of this terminator resulted in a much shorter transcript that was now insensitive to a *upf1* deletion, further showing that the sensitivity of CD-CUTs to NMD is dependent on their long size, and potentially on the lack of extended ORF in the 5′-extension. Strikingly, insertion of the *ADH1t* at positions −771 (B) or −184 (C) resulted in a derepression of *ZRT1* (Figure 4C). The strongest effect was observed for *ADH1t-C*, possibly because this terminator stops all CD-CUT transcription immediately before the *ZRT1* TATA box. In the *ADH1t-B* strain, an increased accumulation of the CD-CUT3 is observed, while this species disappears in the *ADH1t-C* strain. These results show that terminating transcription of the CD-CUTs upstream from the *ZRT1* promoter is sufficient to derepress *ZRT1* in conditions of non-induction. Additionally, insertion of these terminators allowed us to map in further detail the CD-CUTs upstream from *ZRT1*. Based on the hybridization pattern with the different probes (Figure S2A), the effect of the various terminators on their mobility in northern blots (Figure 4C), the approximate architecture of these CD-CUTs is shown in Figure 4D.

**Figure 5:**
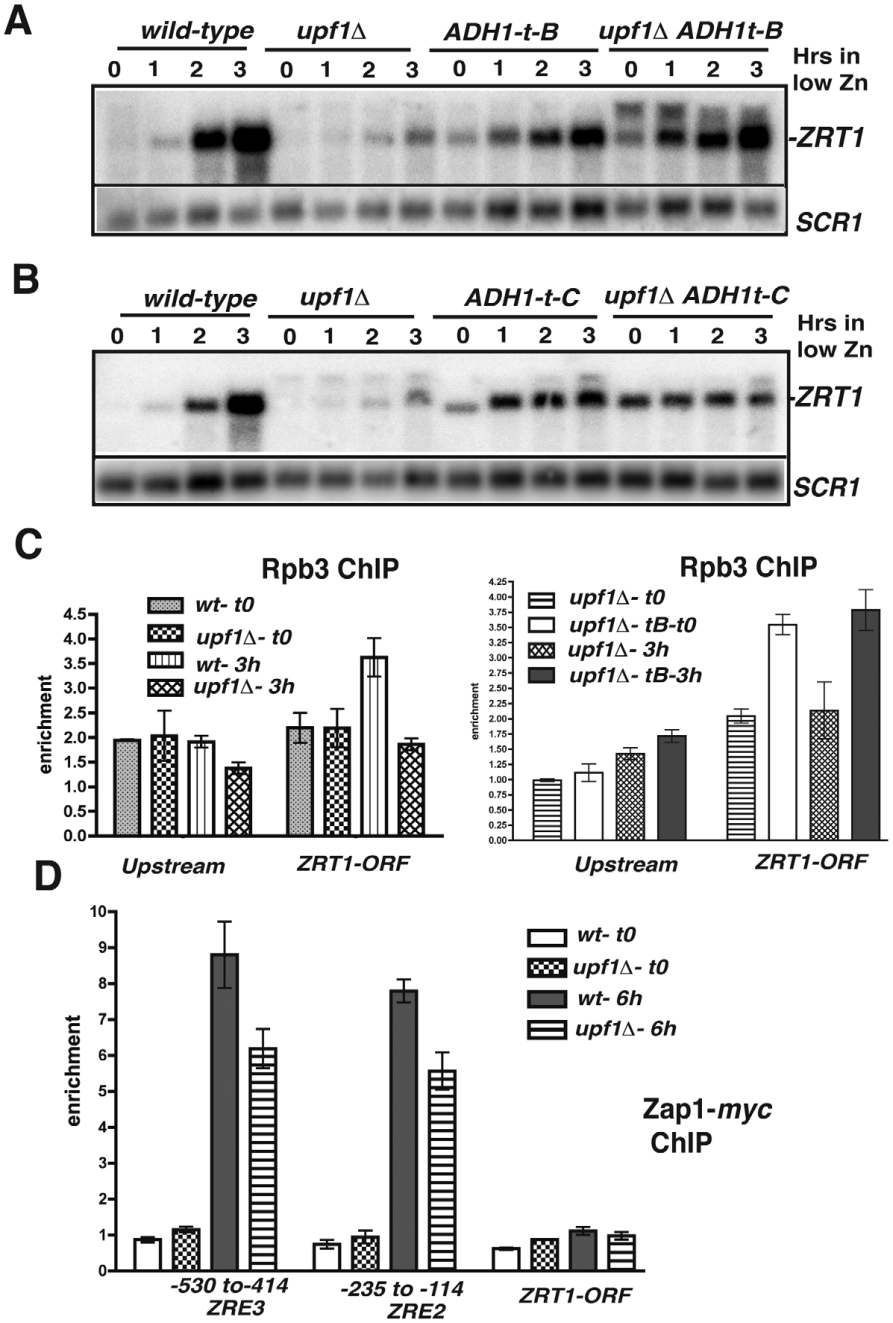
Effects of transcription terminators upstream *ZRT1* on *ZRT1* induction and analysis of RNA Polymerase II and Zap1p occupancies by ChIP. A and B. Effects of the insertion of ADH1 transcriptional terminators at various sites upstream *ZRT1* on *ZRT1* mRNA during zinc depletion in wild-type and *upf1Δ* strains. C. Analysis of RNA polymerase II occupancy in wild-type, *upf1Δ* and *upf1Δ ADH1t*-B strains grown in normal zinc medium (t0) or after a 3 hours shift in low zinc. Shown are normalized qPCR analysis of the different regions from ChIP samples obtained using anti-Rpb3p antibodies normalized to input DNA and to a qPCR product for a control non-transcribed region. D. Analysis of Zap1p occupancy at different sites of the *ZRT1* gene. Legends as in 5C except that a myc-tagged version of Zap1p was used.

Transcription of the zinc regulon genes is activated by binding of the transcriptional activator Zap1p to their promoters during zinc deficiency [28], [38]. The terminator sequence inserted at position 771 is located upstream from the three major Zap1 binding sites (ZRE; [38]), while the terminator sequence inserted at position 184 is inserted downstream from them (Figure 4D). Based on this, we hypothesized that if transcription of the CD-CUTs prevents binding of Zap1p, the two strains containing terminators at the two positions might behave differently during a shift into low zinc conditions. Indeed, insertion of the *ADH1t* at position B derepressed *ZRT1*, and also fully rescued the induction defect of the *upf1Δ* strain (Figure 5A). However strains carrying an insertion of the *ADH1t* at position C failed to induce *ZRT1* in conditions of induction, even in a context of active NMD. This result suggests that the region located between positions B and C, which contains most of the Zap1p binding sites must be accessible for *ZRT1* induction. It is unclear why the strain containing the *ADH1t* at position C failed to induce *ZRT1*, even when NMD is active. It is possible that the higher levels of expression of the *ZRT1* transporter in non-induction conditions resulted in higher cellular zinc levels prior to induction, thus delaying the response. Additionally we cannot rule out that inserting the *ADH1t* at site C might have changed the chromatin structure, and thus perturbed the induction of *ZRT1*.

### Binding of RNA Polymerase II and of the transcriptional activator Zap1p is defective in the NMD mutant *upf1Δ*

To corroborate these results, we studied RNA Polymerase II occupancy in two regions, upstream from *ZRT1* (−1223 to −1123), and within the *ZRT1* ORF (+905 to +1021), using chromatin immunoprecipitation (ChIP) of the Rpb3p subunit. We found slightly above background levels of occupancy of the polymerase in conditions of zinc repletion in both regions in wild-type and *upf1Δ* strains (Figure 5C). Interestingly, Rpb3p occupancy was similar for both strains in the upstream region, indicative of a similar level of transcription of the CD-CUTs. This result further indicates that accumulation of the CD-CUTs in the *upf1Δ* strain is due to a lack of degradation rather than increased transcription. Upon a shift to low zinc medium, Rpb3p occupancy increased for the wild-type strain in the *ZRT1* ORF, but not in the upstream region, reflecting the induction of the *ZRT1* gene. However this increase was not observed in the *upf1Δ* strain, corroborating the results observed by northern analysis. To show that the insertion of the terminator upstream from *ZRT1* rescues the induction defect of the *upf1Δ* strain by allowing polymerase binding, we performed the same analysis by comparing Rpb3p occupancy in the *upf1Δ* and *upf1Δ*-*ADH1t-B* strains. Strikingly, Rpb3p levels were increased upon insertion of *ADH1t* at position B in the *upf1Δ* strain, both in normal zinc medium and after 3 hrs of induction, showing that the derepression of *ZRT1* and the rescue of the induction defects are due to increased RNA Polymerase occupancy.

We also analyzed binding of the transcriptional activator Zap1p by ChIP in wild-type and *upf1Δ* strains using a *myc*-tagged version of Zap1p inserted at the chromosomal locus. We found background levels of Zap1p occupancy to its binding sites (ZREs) in conditions of repression in both strains (Figure 5D). However increased occupancy was observed in the wild-type strain upon a shift to low zinc (Figure 5D). Binding was reduced in the *upf1Δ* mutant, further showing that the accumulation of CD-CUTs perturbs Zap1p binding during *ZRT1* induction. Zap1p enrichment was highly specific, as it was not observed in the *ZRT1* coding region (Figure 5D). Overall the differences in RNA polymerase II and Zap1p occupancies in the *ZRT1* gene are consistent with the results described above by Northern blot, showing that the effects observed in NMD mutants upon accumulation of CD-CUTs are indicative of transcriptional defects of the *ZRT1* gene.

### Overexpression or constitutive activation of transcriptional activators of subtelomeric genes suppresses their induction defect in the *upf1Δ* and *xrn1Δ* strains

**Figure 6:**
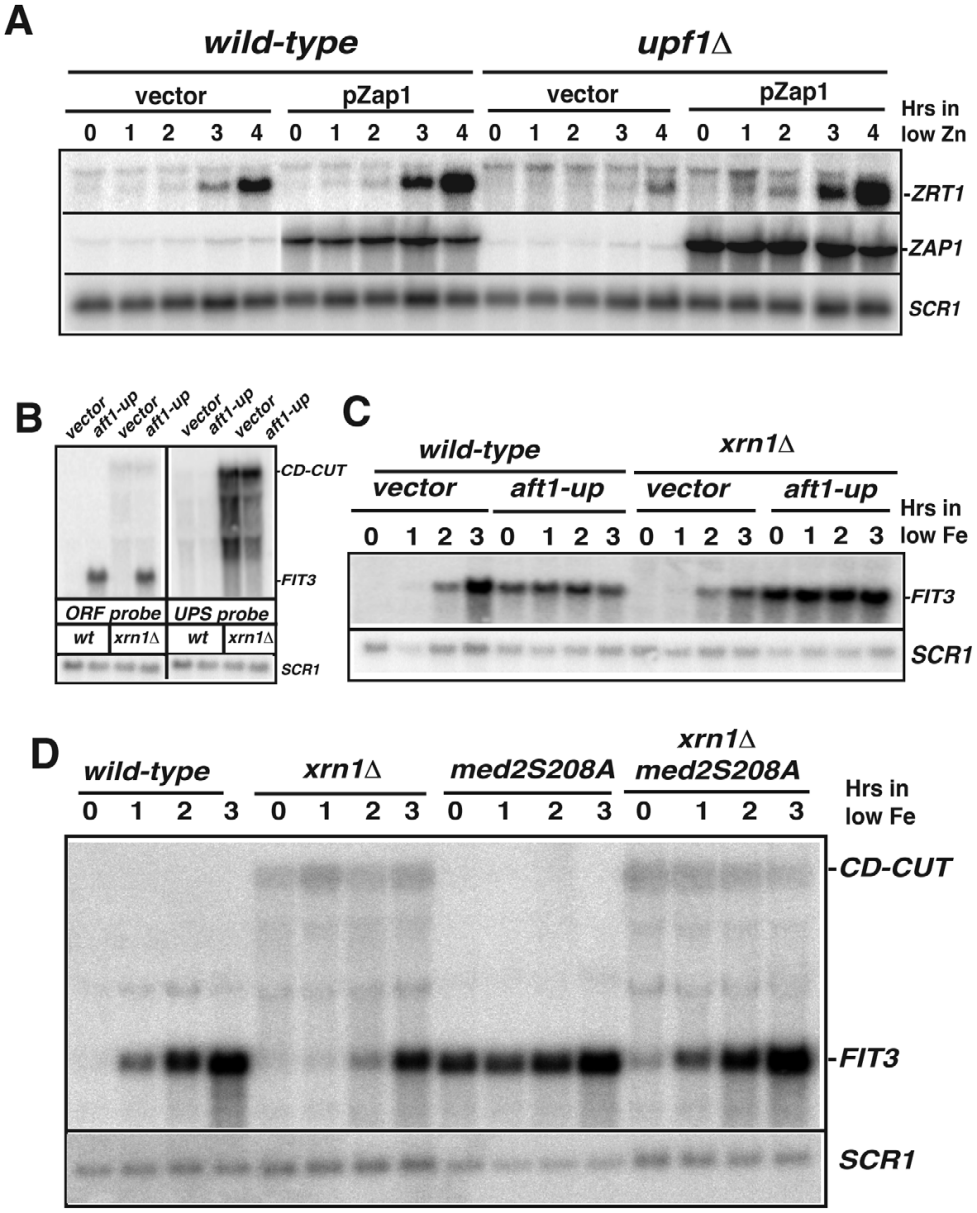
CD-CUT mediated transcriptional repression can be rescued by overexpression or constitutive activation of transcriptional activators. A. Overexpression of Zap1 rescues the *ZRT1* induction defect observed in the *upf1Δ* mutant. Induction of *ZRT1* in wild-type and *upf1Δ* mutant containing an empty vector or a vector overexpressing Zap1p (pZap1) under the control of a *MET25* promoter. The WT or *upf1Δ* stains containing the vector pUG35 or pIT31 were grown in normal zinc conditions (time zero) or after overexpression of Zap1 and shift into low zinc medium. B and C. Expression of the *aft1-up* allele induces *FIT3* expression even in the presence of the *FIT3* CD-CUT. A plasmid expressing the *aft1-up* allele or a control vector were transformed into wild-type and *xrn1Δ* strain, and the levels of *FIT3* mature mRNA or those of the CD-CUT were assessed by northern analysis using an ORF or an upstream (UP) probe, respectively. In panel C, the level of the *FIT3* mRNA was assessed in these strains prior to and after a shift to low iron conditions. D. Analysis of *FIT3* induction in wild-type, *xrn1Δ*, *med2S208A* and double mutant strains.

The previous result showed that binding of the Zap1p activator is deficient in the NMD mutant *upf1Δ* during the low zinc response. If so, we predicted that overexpressing Zap1p might suppress the induction delay in this strain. Indeed, overexpressing Zap1p in the *upf1Δ* mutant was sufficient to rescue *ZRT1* induction to levels comparable to those observed in the wild-type strain (Figure 6A). This result shows that defective binding of Zap1p to the ZREs in the *upf1Δ* mutant can be overcome by overexpressing this activator. To extend these results to another gene induced in different conditions and controlled by a different activator, we monitored the expression of *FIT3* in wild-type and *xrn1Δ* strains expressing *aft1-up*, a constitutively active version of Aft1p (kindly provided by J.Kaplan; [39], [40]. Aft1p is one of the two major transcriptional activators involved in the low iron response [39]. As expected, expression of the *aft1-up* allele resulted in derepression of the *FIT3* mRNA in normal iron conditions in the wild-type strain (Figure 6B). However, similar levels of the mature *FIT3* transcript were observed in an *xrn1Δ* background when the *aft1-up* allele was expressed, showing that the presence of a constitutively activated form of Aft1p can overcome CD-CUT-mediated repression. The accumulation of the *FIT3* CD-CUT was not affected by expression of the *aft1-up* construct (Figure 6B), showing that this effect was not due to a decrease of expression of CD-CUT. We also monitored *FIT3* induction in these strains upon a shift to low iron conditions (Figure 6C). Interestingly, *FIT3* levels did not increase in the *aft1-up* strain upon a shift to low iron conditions (Figure 6C). However *FIT3* accumulation was higher in the *xrn1Δ aft1-up* double mutant, possibly because of a reduced degradation of the *FIT3* mRNA in the absence of Xrn1p.

### Accumulation of CD-CUTs abolishes the derepression of *FIT3* induced by a Mediator component mutation

To investigate the specificity of the effects described above, we searched for conditions in which the induction of *ZRT1* or *FIT3* was uncoupled from activation by their transcriptional activators. Mutation of the Med2p tail component of the Mediator complex into a non-phosphorylated isoform (*med2-S208A*) was shown to result in a constitutive expression of *FIT3* [41]. This observation led us to investigate the effect of the accumulation of the *FIT3* CD-CUT on the derepression of *FIT3* induced by this Mediator component mutation. We inactivated Xrn1p in a strain carrying the *med2-S208A* mutation (kind gift of F.Holstege) and analyzed the expression of *FIT3*. *FIT3* derepression was observed in the *med2-S208A* mutant grown in normal medium (Figure 6D; time zero), in agreement with previous findings [41], but this mutant did not exhibit any further induction in low iron until 3 hours after the shift. Strikingly, inactivating Xrn1p in the *med2-S208A* strain abolished the derepression of *FIT3* observed in the *med2-S208A* strain (Figure 6D). However the *xrn1Δmed2-S208A* mutant strain was not as defective for induction as the *xrn1Δ* strain, since the double mutant showed kinetics of *FIT3* induction comparable to the wild-type strain. The result obtained in normal iron conditions (time zero, Figure 6D) shows that the accumulation of the *FIT3* CD-CUT can inhibit the activation of *FIT3* that results from a mediator component mutation. These results contrast with the result observed previously with the *aft1-up* mutation, which can activate expression of *FIT3* even when CD-CUTs accumulate due to the inactivation of Xrn1p. Taken together, these results suggest that the function of the *FIT3* and *ZRT1* CD-CUTs is to prevent the premature binding of the RNA polymerase or of transcriptional activators such as Zap1 and Aft1p when these genes are transcriptionally repressed.

## Discussion

Stable non-coding RNAs that can be detected without perturbations of the RNA degradation machinery have been shown to accumulate near many genes [42]–[44]. Here we show that strains defective for NMD accumulate 5′-extended forms (CD-CUTs) of many subtelomeric genes, some of which are involved in zinc and iron uptake and homeostasis. The accumulation of CD-CUTs is observed for a large number of genes located in subtelomeric regions in NMD mutants (Figure 1; Table S1). NMD also degrades the upstream sense ncRNA *ICR1* that regulates the *FLO11* gene ([8]; Figure S1B). Therefore CD-CUTs degraded by NMD are not exclusively involved in regulating metal homeostasis genes. Given the number of transcripts for which we detected a potential accumulation of CD-CUTs in NMD mutants by tiling arrays (Table S1), and the fact that extended forms of *SRG1*, which regulate *SER3* transcription can be degraded by NMD [13], we speculate that cryptic upstream sense transcription might be used more widely than previously thought. However most of these transcripts are normally undetectable or present at very low levels because of active NMD.

**Figure 7:**
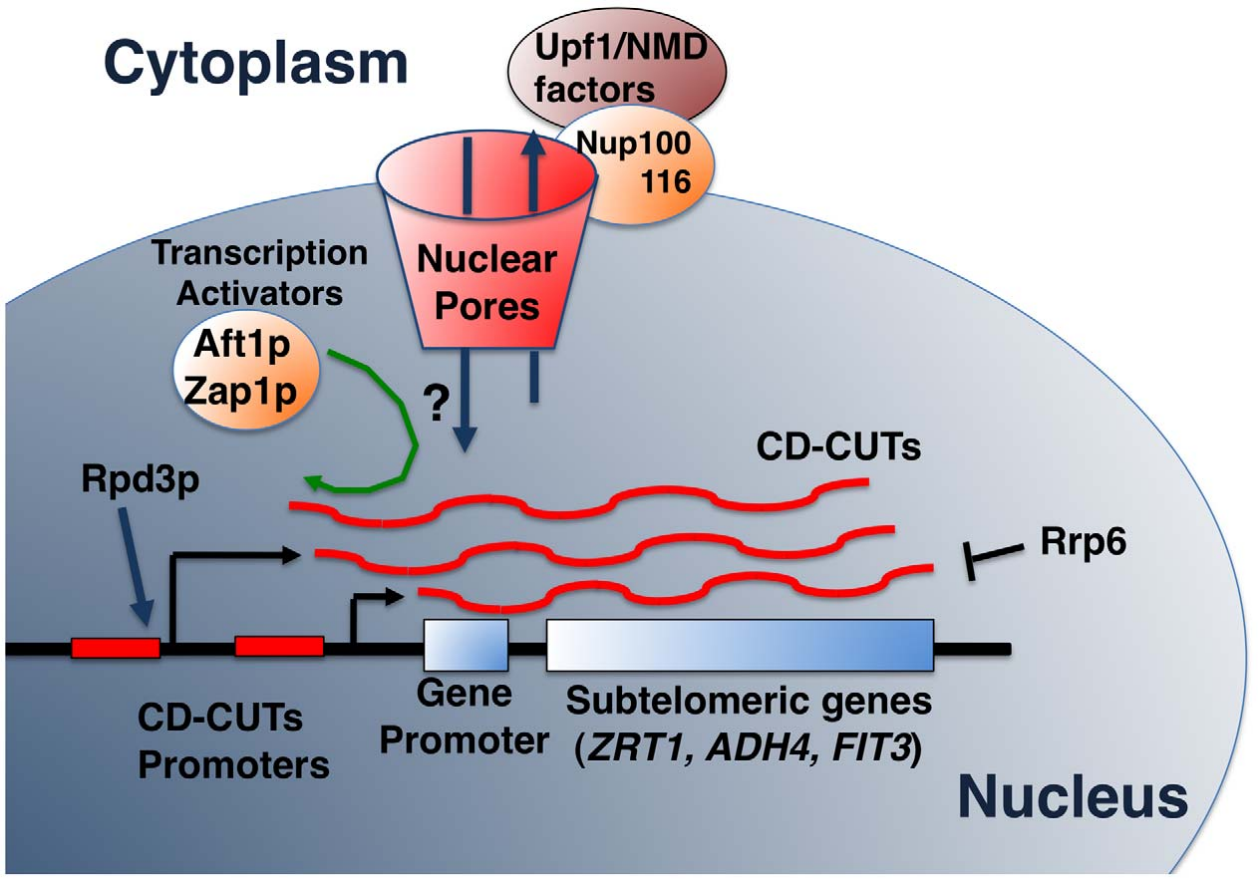
Model of biogenesis, action, and degradation of CD-CUTs. The model shows the degradation of CD-CUTs by the NMD pathway, potentially localized at the vicinity of the nuclear pore due to the anchoring by the Nup100/116 nucleoporins [50]. Hypothetical retrograde transport of CD-CUTs is shown by a question mark but would explain the increased repression of subtelomeric genes in the absence of cytoplasmic degradation in NMD mutants.

CD-CUTs are clearly degraded by NMD, as shown by their extended half-life in the absence of Upf1p (Figure 2F, 2G) and because they are no longer stabilized by a *upf1* deletion when a long ORF is inserted in their place (Figure S5B). CD-CUTs are likely recognized as NMD substrates because of the lack of extended ORFs in the 5′-regions upstream from the natural ORF. This might result in random translation initiation in the 5′-region, followed shortly by a stop codon, resulting in recognition of a *faux*/extended 3′-UTR [45], thus targeting them to NMD. In support of this model, terminating transcription of the main *ZRT1* CD-CUT in a manner that results in a shorter transcript renders it insensitive to a *upf1* deletion (Figure 4C, *adh1t-A* strain). CD-CUTs accumulating in NMD-deficient strains are different from the CUTs accumulating in nuclear exosome or TRAMP complex mutants [11], [46], [47]. CD-CUTs are much larger than CUTs, and most of them extend within the open-reading frames to terminate at or near the site of normal 3′ processing of the ORF mRNAs (Figure 1 and Figure 2, Figure S2, Figure 4D). Thus, both the nuclear exosome and cytoplasmic NMD degradation machineries are used to regulate gene expression but act on different sets of promoter-associated unstable RNAs. However, the two pathways can sometimes intersect, for example, in the case of the *ADH4* CD-CUT, which is efficiently degraded only when both NMD and the nuclear exosome is inactivated (Figure 2; Figure 7).

### How do CD-CUTs mediate transcriptional repression?

The precise mechanism by which CD-CUTs mediate transcriptional repression is not fully understood. Accumulation of CD-CUTs in NMD mutants negatively interferes with production of the normal transcripts and with RNA polymerase II and transcriptional activator binding (Figure 5, Figure 6, Figure 7). We do not know whether acting in *cis* is strictly required, which would be consistent with an *SRG1*-like transcriptional interference model [3]. We tried to express the *ZRT1* CD-CUTs from a plasmid to test for the possibility of a *trans* effect, but could not detect any reproducible effect on *ZRT1* induction (data not shown). A recent study showed that transcription of the sense upstream ncRNA *ICR1* mediates transcriptional control of the subtelomeric *FLO11* gene [8]. We found that the region upstream from the *FLO11* gene encoding *ICR1* shows elevated RNA levels in the *upf1Δ* strain (Figure S6). Like *ICR1*, expression of the *ZRT1* CD-CUT is under the control of the histone deacetylase Rpd3p (Figure 4 and [8]). Based on these similarities, it is possible that the mechanisms of transcriptional control of the *FLO11* gene mediated by *ICR1* described in [8] may be applicable to the action of the CD-CUTs that control other subtelomeric regions.

The experiments in which we inserted transcription terminators upstream from *ZRT1* do not allow us to differentiate between the *cis* and *trans*-acting models for CD-CUTs. Insertion of the terminator at position B relieves repression and allows the *upf1Δ* strain to induce *ZRT1* in low zinc conditions (Figure 5). However in this strain, the CD-CUTs terminate before the Zap1 binding sites, so the results could be interpreted either way (transcriptional interference or *trans*-acting). Insertion of the terminator at position C relieves repression, but also inhibits *ZRT1* induction even when NMD is active (Figure 5). Thus, we cannot conclude whether the CD-CUTs act in *trans* or are only the product of transcription that generates transcriptional interference. Because NMD mutants show higher CD-CUTs levels without a higher level of RNA Polymerase in the CD-CUT transcribed region (Figure 5C) and also result in stronger repression, we favor the hypothesis that these RNAs act in *trans*. However, further work is required to fully prove this point. Another unanswered question is to understand how the transcriptional machinery overcomes CD-CUT mediated repression in conditions of induction. It is possible that CD-CUT transcription is decreased in these conditions, but neither northern analysis nor the ChIP data seem to indicate that this is the case. Another alternative is that activation of the transcriptional activators is so potent during induction that it can overcome CD-CUTs mediated repression, even if the level of transcription of CD-CUTs does not change. The results obtained with the Zap1p overexpression or the constitutive Aft1p allele strains (Figure 6) are consistent with this model.

### How does a cytoplasmic degradation pathway influence nuclear transcription?

One of the paradoxes raised by our observations is that 5′-extended species of subtelomeric genes are degraded by NMD, which is a cytoplasmic degradation pathway (Figure 7), yet, these CD-CUTs mediate transcriptional repression, and must therefore be localized to the nucleus if they mediate repression. Interestingly, many subtelomeric genes exhibit a perinuclear localization in *S.cerevisiae* [48]. In addition, connections have been made between nuclear activation of genes and localization at the periphery of the nuclear envelope near the nuclear pores (reviewed in [49]). Finally, Upf1p has been shown to interact with two nucleoporins localized on the outer side of the nuclear envelope, Nup100p and Nup116p ([50]; Figure 7), suggesting that at least part of the NMD process might occur at the vicinity of the nuclear envelope (Figure 7). Therefore if both subtelomeric genes and NMD components are localized close to the nuclear envelope but on opposite sides of the nuclear pores, the physical distance between the sites of transcription and action of CD-CUTs, and their site of degradation might be closer than thought from just considering the nuclear/cytoplasmic distribution (Figure 7). This would possibly allow retrograde transport of these CD-CUTs from their site of degradation to their site of action, and would allow a regulation of transcription by ncRNAs primarily degraded in the cytoplasm (Figure 7). It is also possible that CD-CUTs might be degraded when they emerge out of the nuclear pore complex, and that failure to degrade induces an increase of their nuclear localization, explaining a higher level of repression in NMD mutants. A better analysis of the mechanisms of nuclear/cytoplasmic trafficking of these CD-CUTs will ultimately allow a full understanding of their mechanisms of action.

### Conserved functions for ncRNAs and NMD in the control of the expression of metal homeostasis genes

Despite these unanswered questions, our results have uncovered a novel function for NMD in controlling the accumulation of transcripts that negatively interfere with transcription of genes involved in zinc and iron homeostasis. Previous work has shown that upstream ncRNAs are involved in controlling gene expression related to metals homeostasis. It was shown that Zap1p activates the transcription of ncRNAs which mediate the repression of zinc-dependent alcohol dehydrogenases by transcriptional interference during zinc deficiency [51]. However it is unclear whether or not these ncRNAs are targeted by NMD, in a manner similar to CD-CUTs that regulate *ZRT1*, *ADH4* and *FIT3*. A potential transcriptional interference mechanism involving long unstable upstream sense ncRNA has also been described in *Chlamydomonas* during copper deficiency [52], suggesting that this mechanism has been conserved during evolution to contribute generally to metal homeostasis genes regulation. Thus, there seems to be prevalent use of ncRNA transcription to control gene expression during metals homeostasis in different organisms. In addition to the potential crosstalk with transcription described here, NMD was also recently shown to regulate Mg^++^ cellular levels [23] by degrading the transcript encoding the main Mg^++^ transporter. The fact that this RNA surveillance system is so intimately implicated in the regulation of metals homeostasis in general might be linked to the prevalence of these metals in the ribosome and in their function of translation and in its fidelity [23], possibly revealing another layer of co-evolution between NMD and translation.

## Materials and Methods

### Yeast strains and media

Most strains were derived from BY4741 or 4742 (Open Biosystems). Strains in which the *GFP-HIS* cassette in the upstream region of *ZRT1* or *ADH4* gene were obtained by homologous recombination [36]. The strain carrying the deletion of the region upstream *FIT3* and the strains containing the terminator insertions were obtained by *delitto perfetto* [53]. Double mutants in which the *UPF1* or *XRN1* genes were knocked out were obtained by direct disruption of these genes in other mutant strains, as described [21]. Insertion of the myc-tag for Zap1p was performed as described [36].

Strains were grown in conditions of non-induction in either YPD or Synthetic Complete medium (SC) supplemented with 2 mM ZnCl_2_. Growth in condition of low zinc gene induction was performed in either a Chelex-treated synthetic complete medium (CSC) or a SC medium containing 1 mM EDTA, pH adjusted to 4.4 with 20 mM citrate. CSC was prepared as described [28] except that all amino acid required were added and pH adjusted to 4.4. Growth in conditions of low iron gene induction was performed by adding BPS chelator as described [31]. Strains were grown in YPD (pre-low iron shifts) or SC+2 mM Zn (pre-low zinc shifts) until OD_600_ = 0.5, washed twice in sterile water, and shifted into YPD medium with BPS (low iron shift) or SC+EDTA medium (low zinc shift) for the indicated times. For the experiments including overexpression of Zap1p, WT or *upf1Δ* strains containing the vector pUG35 or pIT31 (see below) were grown in SC medium without Uracil (SC-URA) supplemented with 2 mM Zn. At OD_600_ = 0.45, cells were washed twice in sterile water and shifted in SC-URA without methionine (SC-URA-MET) supplemented with 2 mM Zn for 2 hours to overexpress Zap1p prior to zinc starvation. After 2 h, cells were washed twice in sterile water and maintained in log phase in SC-URA-MET medium containing 1 mM EDTA. Kinetics of induction were performed as described above.

### Plasmids

A PCR product corresponding to the *ZAP1* gene was generated from genomic DNA with primers containing the restriction sites *ClaI* and *SalI* and inserted in the vector pUG35 digested by the same restriction enzymes. After transformation and amplification in *E. coli*, the plasmid (pIT31) was confirmed by sequencing. The *aft1-up* expression plasmid was obtained from J.Kaplan (U.of Utah).

### RNA analysis

Tiling Arrays used in this study were described previously [21], [31] and are accessible in the GEO database (accession number GSE11621). Northern blot hybridization analysis was performed as previously described [21], [31]. All riboprobes were synthesized with the T3 MAXIscript kit (Ambion). Riboprobes were hybridized at 67°C except for the *ADH4* ORF probe (65°C).

### Chromatin Immunoprecipitation (ChIP)

ChIPs using anti-Rpb3 RNA polymerase II subunit and a myc-tagged version of Zap1p inserted at the chromosomal locus were performed as described [54], [55].

## Supporting Information

Figure S1 Tiling array profiles of *ZPS1* and *FLO11*. A. Tiling array profile in the region of the *ZPS1* gene. Shown is the log_2_ ratio of signal detected for the *upf1Δ* strain divided by the signal for the wild-type strain. B. Tiling Array profile of the *upf1Δ* mutant compared to the wild-type strain in the right subtelomeric region of Chr IX containing the *FLO11* gene. The *FLO11* gene is localized on the Crick strand (transcribed right to left) from positions 393,672 to 389,569. The increase of signal in the *upf1Δ* mutant upstream from the *FLO11* gene is indicative of higher levels of the *ICR1* ncRNA controlling *FLO11*. Note that the neighbor genes *MRS1* and *SEC11* located in region 397,000–399,000 are not affected by the *upf1Δ* deletion.
(TIF)

Figure S2 Mapping of the CD-CUTs of *ZRT1* (A) and *ADH4* (B) genes by northern blot analysis. The *ZRT1* probes cover the following nucleotides: Probe 1: −2410 to −2089; Probe 2: −800 to −211; Probe 3: −257 to −84; Probe 4: +931 to +1131; Probe 5: +1141 to +1341 (downstream from the ORF).
(TIF)

Figure S3 Analysis of zinc regulon genes induction in NMD mutants. A. Kinetics of induction of zinc regulon genes in wild-type, *upf2Δ* and *upf3Δ* strains after a shift to a medium lacking zinc. Gene induction was monitored by northern blot using probes hybridizing to the corresponding genes. *SCR1* was used as a loading control. B. Analysis of *ZAP1* mRNAs levels in wild-type and *upf1Δ* strains. *ZAP1* mRNA levels were analyzed by northern blot from cells grown in SC+2 mM Zn (time zero), or after a shift to SC+EDTA (low Zn). An RNA sample from a *zap1Δ* strain was included as a negative control for the detection of the ZAP1 mRNA.
(TIF)

Figure S4 Kinetics of *FIT3* induction in strains lacking Xrn1p, Upf1p or in the double mutant *xrn1Δrat1-1*. Shown is a northern blot analysis of *FIT3* expression in the indicated strains using a probe complementary to *FIT3*.
(TIF)

Figure S5 Insertion of GFP cassettes upstream from *ZRT1* and *ADH4*. A. Schematic representation of the *ZRT1* and *ADH4* genes and location of the *GFP*-*HIS3* cassettes deletions/insertions (UP1 and UP2, UP). The dashed lines represent the regions replaced by the cassettes. B. GFP mRNA levels analyzed by northern blot in the different insertion strains. Levels were normalized to *SCR1*. A strain without GFP insertion was included as negative control. C. Effects of the deletion of the upstream region and of the insertion of a *GFP-HIS3* cassette on the kinetics of induction and shutoff of *ZRT1*. Insertion of the *GFP-HIS3* cassette upstream from *ZRT1* (*zrt1-up1*) results in a three-fold increase in the *ZRT1* mRNA peak in the *upf1Δ* strain during zinc deficiency. *ZRT1* induction was faster in the strain carrying this insertion compared to the wild-type, as shown by the amount of the *ZRT1* mRNA expressed after 30 to 120 minutes. After 4 hours of induction, the kinetics of disappearance of *ZRT1* upon shifting back to zinc-containing medium was also monitored in these strains (+zinc), and samples were harvested at the indicated times after addition of zinc to the medium. RNAs extracted from all four strains were loaded on the same gel and analyzed on the same membranes exposed to the same times, but each strain is shown as a separate panel for clarity and space purposes.
(TIF)

Figure S6 Kinetics of *ZRT1* induction in wild-type, *hda2Δ hda3Δ*, *upf1Δ*, *hda2Δupf1Δ* and *hda3Δupf1Δ* strains.
(TIF)

Table S1 List of *S.cerevisiae* ORFs for which 5′ extended species were observed by tiling microarrays in the *upf1Δ* and/or *xrn1Δ*strains.
(XLS)
