# Supplementary figures and images for: Cryptic Transcription Mediates Repression of Subtelomeric Metal Homeostasis Genes
Source: PLoS Genet. 2011 Jun 30;7(6):e1002163. doi: 10.1371/journal.pgen.1002163 (PMC3128112; doi:10.1371/journal.pgen.1002163)

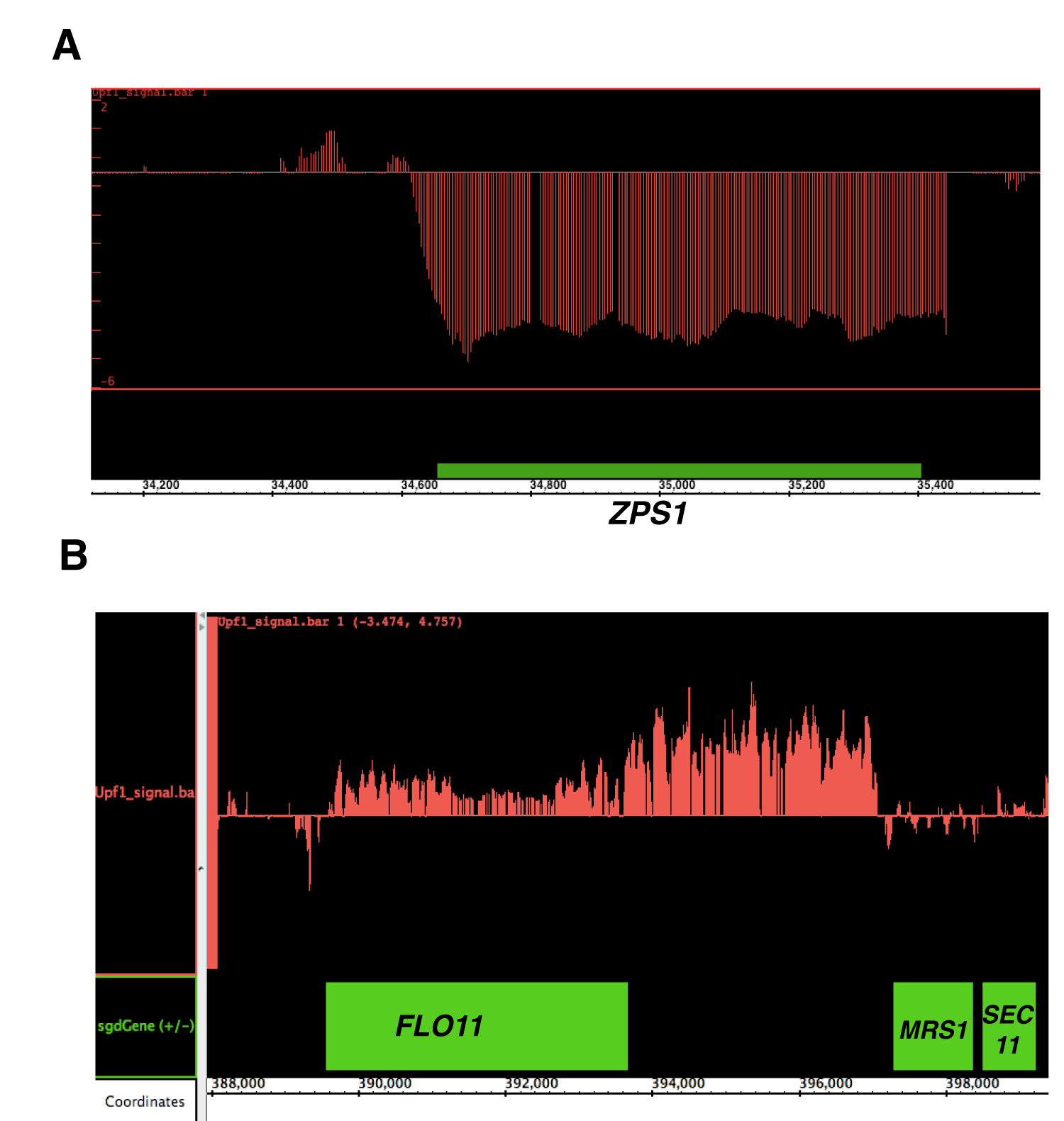

Supplement: Figure S1 — Tiling array profiles of ZPS1 and FLO11. A. Tiling array profile in the region of the ZPS1 gene. Shown is the log2 ratio of signal detected for the upf1Δ strain divided by the signal for the wild-type strain. B. Tiling Array profile of the upf1Δ mutant compared to the wild-type strain in the right subtelomeric region of Chr IX containing the FLO11 gene. The FLO11 gene is localized on the Crick strand (transcribed right to left) from positions 393,672 to 389,569. The increase of signal in the upf1Δ mutant upstream from the FLO11 gene is indicative of higher levels of the ICR1 ncRNA controlling FLO11. Note that the neighbor genes MRS1 and SEC11 located in region 397,000–399,000 are not affected by the upf1Δ deletion. (TIF) [file pgen.1002163.s001.tif]

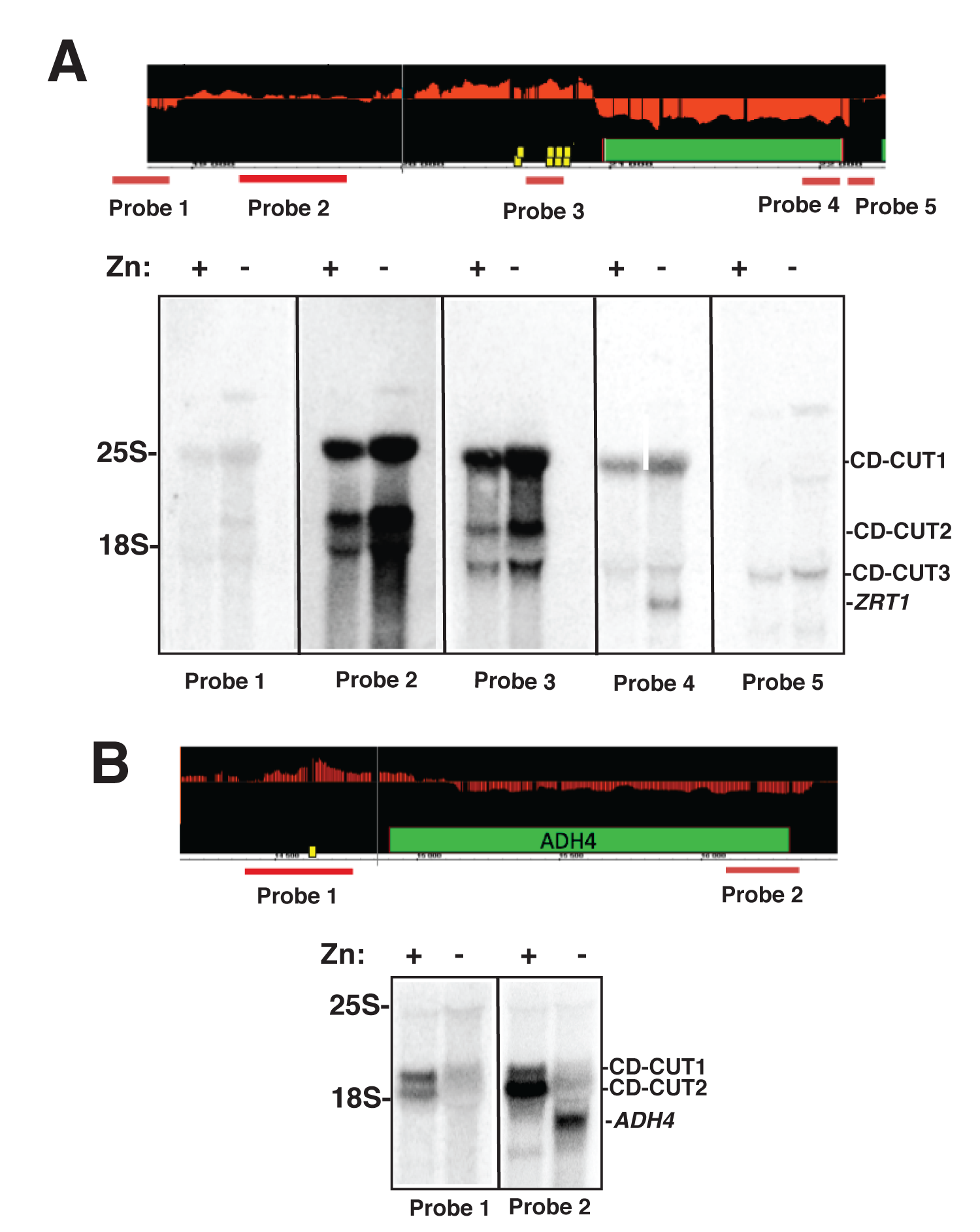

Supplement: Figure S2 — Mapping of the CD-CUTs of ZRT1 (A) and ADH4 (B) genes by northern blot analysis. The ZRT1 probes cover the following nucleotides: Probe 1: −2410 to −2089; Probe 2: −800 to −211; Probe 3: −257 to −84; Probe 4: +931 to +1131; Probe 5: +1141 to +1341 (downstream from the ORF). (TIF) [file pgen.1002163.s002.tif]

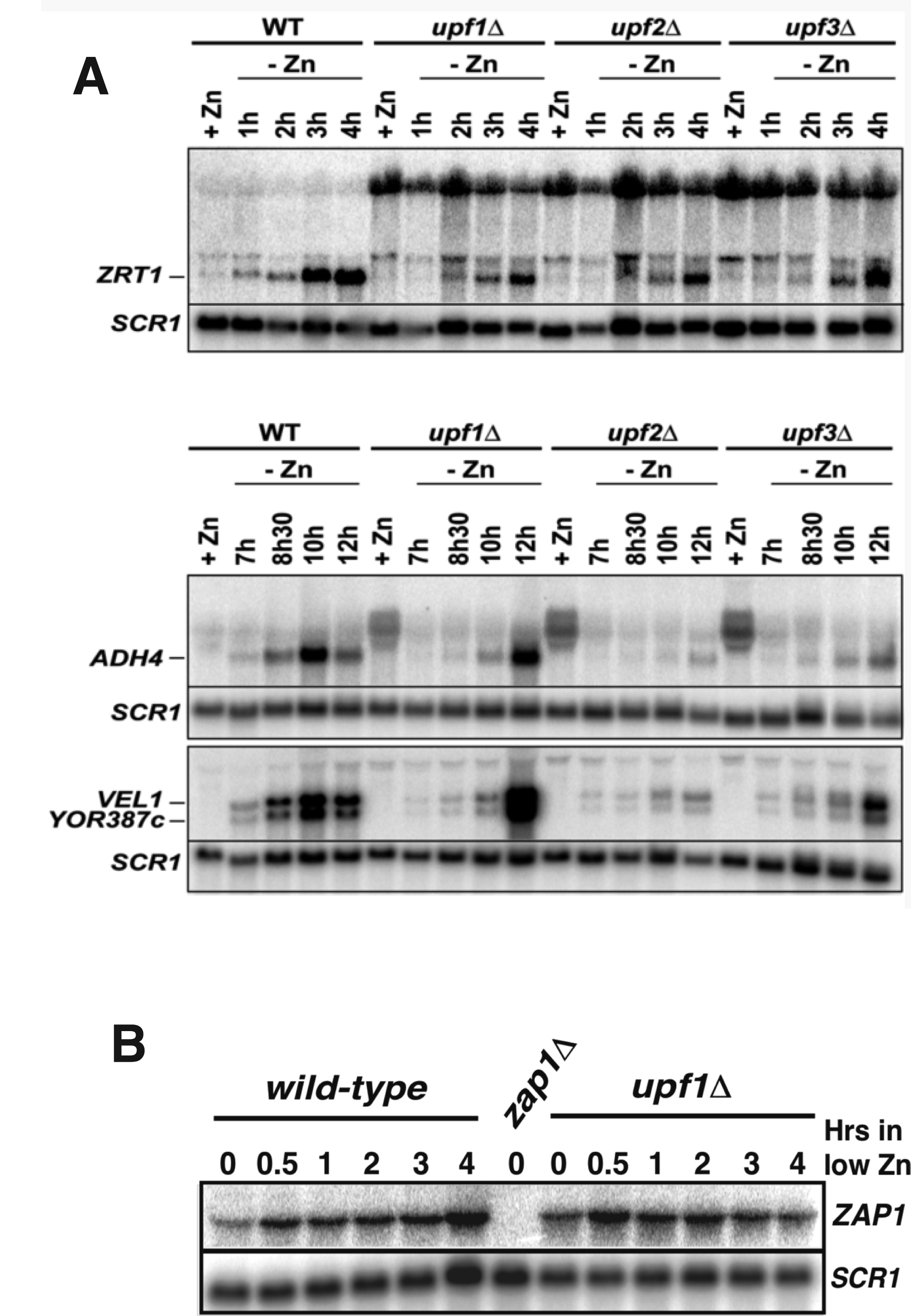

Supplement: Figure S3 — Analysis of zinc regulon genes induction in NMD mutants. A. Kinetics of induction of zinc regulon genes in wild-type, upf2Δ and upf3Δ strains after a shift to a medium lacking zinc. Gene induction was monitored by northern blot using probes hybridizing to the corresponding genes. SCR1 was used as a loading control. B. Analysis of ZAP1 mRNAs levels in wild-type and upf1Δ strains. ZAP1 mRNA levels were analyzed by northern blot from cells grown in SC+2 mM Zn (time zero), or after a shift to SC+EDTA (low Zn). An RNA sample from a zap1Δ strain was included as a negative control for the detection of the ZAP1 mRNA. (TIF) [file pgen.1002163.s003.tif]

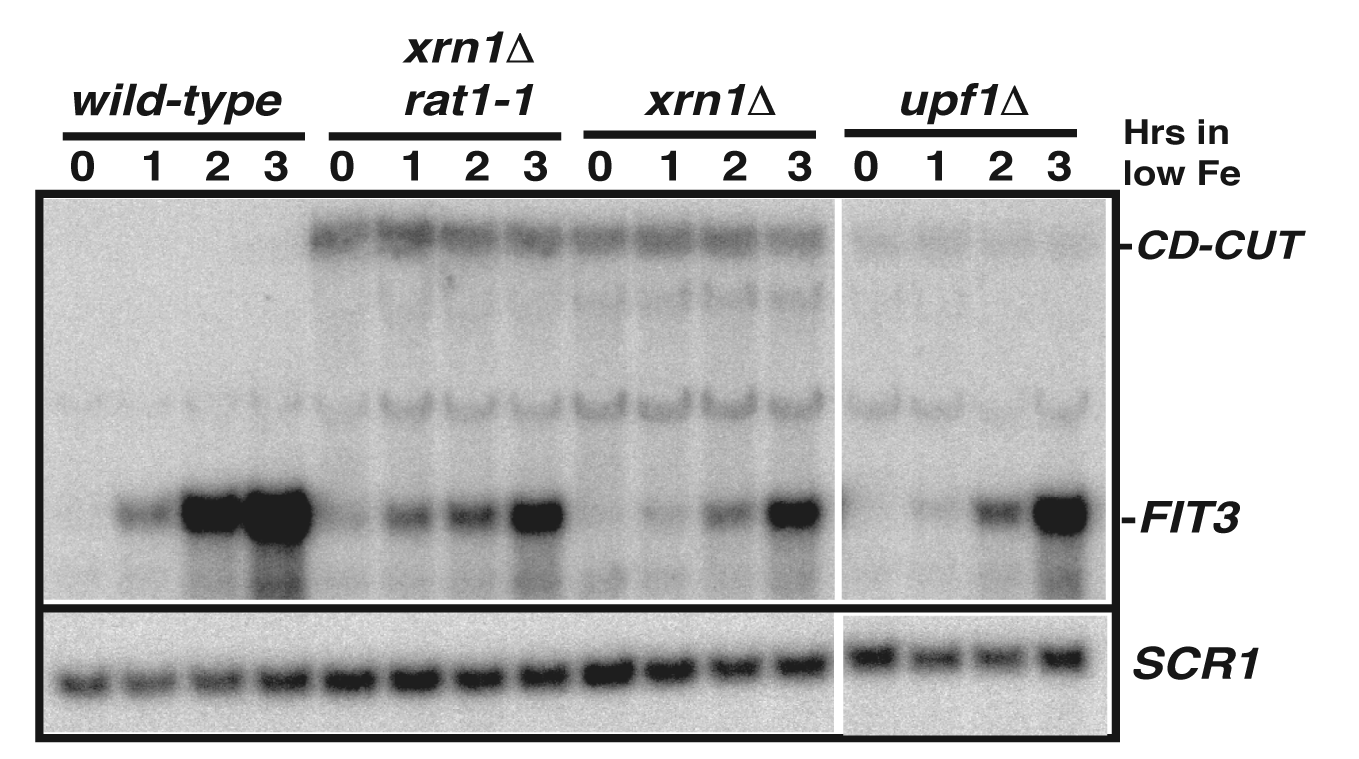

Supplement: Figure S4 — Kinetics of FIT3 induction in strains lacking Xrn1p, Upf1p or in the double mutant xrn1Δrat1-1. Shown is a northern blot analysis of FIT3 expression in the indicated strains using a probe complementary to FIT3. (TIF) [file pgen.1002163.s004.tif]

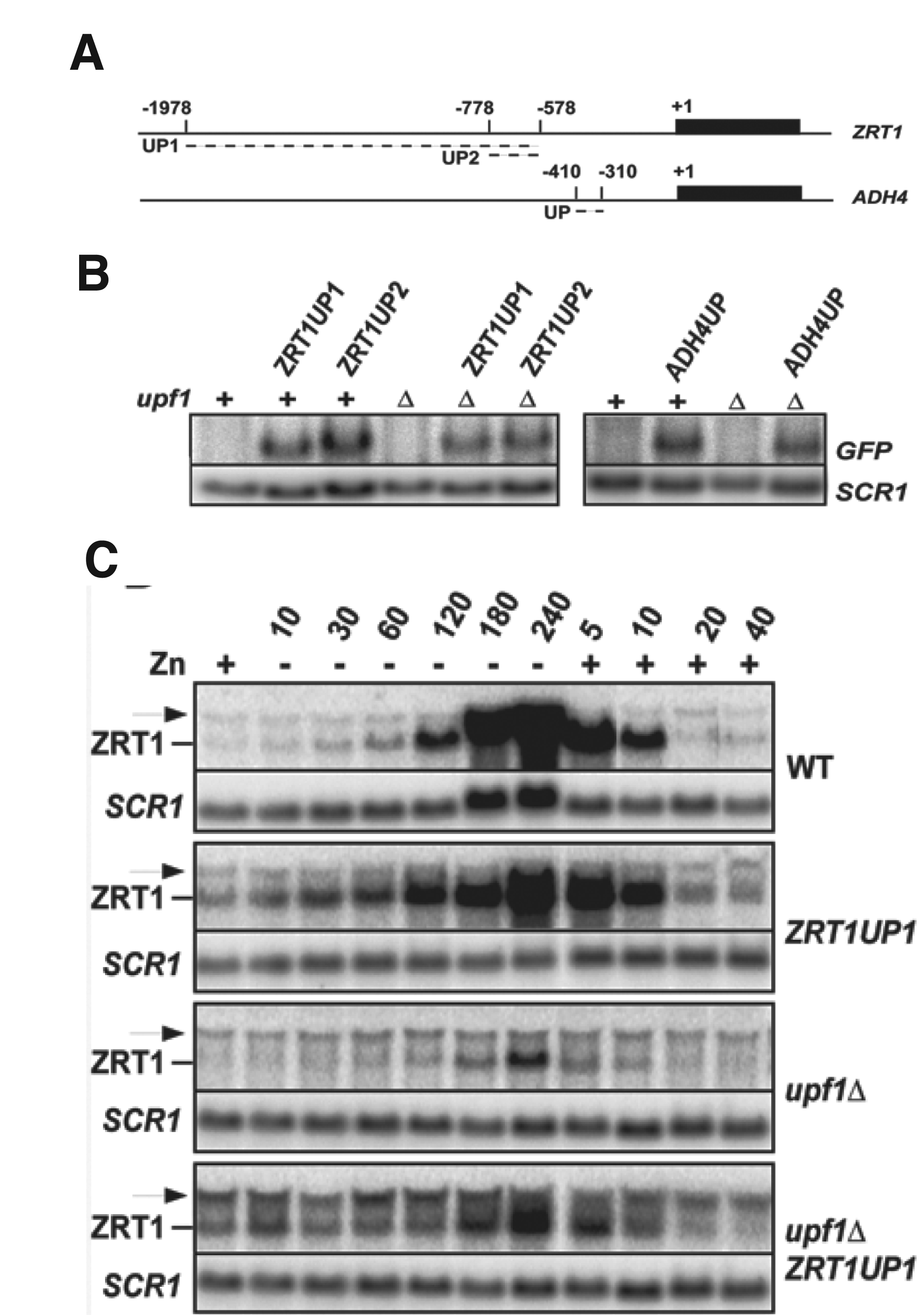

Supplement: Figure S5 — Insertion of GFP cassettes upstream from ZRT1 and ADH4. A. Schematic representation of the ZRT1 and ADH4 genes and location of the GFP-HIS3 cassettes deletions/insertions (UP1 and UP2, UP). The dashed lines represent the regions replaced by the cassettes. B. GFP mRNA levels analyzed by northern blot in the different insertion strains. Levels were normalized to SCR1. A strain without GFP insertion was included as negative control. C. Effects of the deletion of the upstream region and of the insertion of a GFP-HIS3 cassette on the kinetics of induction and shutoff of ZRT1. Insertion of the GFP-HIS3 cassette upstream from ZRT1 (zrt1-up1) results in a three-fold increase in the ZRT1 mRNA peak in the upf1Δ strain during zinc deficiency. ZRT1 induction was faster in the strain carrying this insertion compared to the wild-type, as shown by the amount of the ZRT1 mRNA expressed after 30 to 120 minutes. After 4 hours of induction, the kinetics of disappearance of ZRT1 upon shifting back to zinc-containing medium was also monitored in these strains (+zinc), and samples were harvested at the indicated times after addition of zinc to the medium. RNAs extracted from all four strains were loaded on the same gel and analyzed on the same membranes exposed to the same times, but each strain is shown as a separate panel for clarity and space purposes. (TIF) [file pgen.1002163.s005.tif]

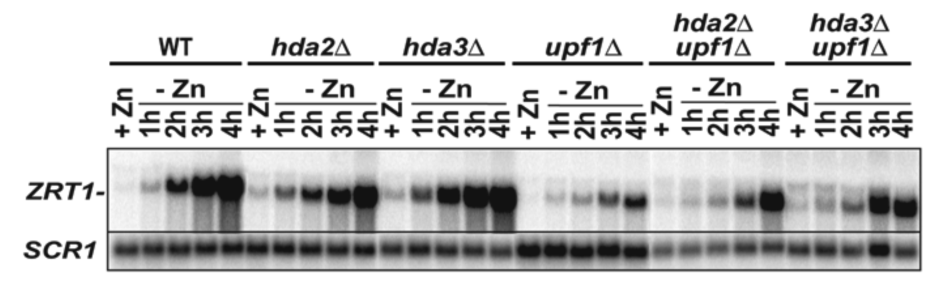

Supplement: Figure S6 — Kinetics of ZRT1 induction in wild-type, hda2Δ hda3Δ, upf1Δ, hda2Δupf1Δ and hda3Δupf1Δ strains. (TIF) [file pgen.1002163.s006.tif]
